# Selected Biochemical Properties of Medicinal Plant (*Urtica dioica* L.) Leaves in Relation to the Enzymatic Activity of Soils Exposed to the Impact of Road Traffic

**DOI:** 10.3390/molecules30214298

**Published:** 2025-11-05

**Authors:** Joanna Lemanowicz, Iwona Jaskulska

**Affiliations:** 1 Division of Biochemistry, Faculty of Medicine, Bydgoszcz University of Science and Technology, Bernardyńska 6 St., 85-029 Bydgoszcz, Poland; 2 Department of Agronomy and Food Processing, Faculty of Agriculture and Biotechnology, Bydgoszcz University of Science and Technology, 7 Kaliskiego St., 85-796 Bydgoszcz, Poland; iwona.jaskulska@pbs.edu.pl

**Keywords:** antioxidant activity, alkaline and acid phosphatase, APTI, ascorbic acid, *β*-glucosidase, catalase, chlorophylls, dehydrogenases, proteases, soil, superoxide dismutase

## Abstract

This study examined the impact of distance from the road traffic on soil enzymatic activity, which we used as a tool to assess the relationship between soil and common nettle (*Urtica dioica* L.) used in herbalism and phytotherapy. A section of national road No. 10 (DK10) was selected for the study. Soil and common nettle leaf samples were collected from locations 5 m, 15 m, 25 m, and 100 m away from the road traffic and a control location (C). The activity of catalase (CAT), dehydrogenases (DEH), alkaline phosphatase (AlP), acid phosphatase (AcP), protease (PRO) and β-glucosidase (BG) was examined in the soil. Soil quality indices (RCh, RS, AlP/AcP, GMea, TEI) were calculated based on the enzyme activity results. The leaves of common nettles were tested for chlorophylls a and b (Chl a and b), carotenoids (Car), ascorbic acid (AAC), pH, relative water content (RWC), catalase (CATp) and superoxide dismutase (SOD) activity. Based on the values of Chl a+b, Car, pH, and RWC, the air pollution tolerance index (APTI) was calculated. The activity of the tested enzymes was statistically lowest in soil collected 5 m from traffic compared to the control (C), which was also confirmed by the results of the enzymatic soil quality indicators. In the case of CAT, AlP, AcP, and BG, based on the coefficient of determination (R2), it was found that over 70% of the variability of these enzymes was related to the distance from the road. It was found that the content of Ch a and b, Car, AAC, RWC, and pH was also lowest in soil 5 m away, whereas the activity of the antioxidant enzymes CATp and SOD was highest at this point. The ATPI values determined in common nettle leaf samples collected from locations 5 m, 15 m, 25 m, and 100 m from the road traffic were sensitive to pollution. The results indicate that the distance from the road strongly influenced the changes in the parameters studied. The enzymatic properties of the soil and selected biochemical parameters of common nettle leaves were similar at locations 15 m and 25 m, as well as 100 m and the control. The results of the enzymatic soil quality indicators show that soil 5 m from the road traffic is subject to degradation, and the nettles growing in this location are sensitive to road pollution. Therefore, it is not recommended to collect common nettle leaves from this location for medical or cosmetic purposes.

## 1. Introduction

Common nettle (*Urtica dioica* L.) is a synanthropic plant that adapts to living in environments heavily altered by humans. It is a ruderal plant but has recently gained more attention due to its health-promoting properties [1]. Stinging common nettle is found in areas such as households, near lakes and rivers, clearings, pastures, and degraded areas. However, the possibility of heavy metal accumulation may impair the quality of wild-growing plant raw materials [2]. Therefore, in recent years, there has been a need to cultivate nettles on a larger scale to meet the growing demand for their leaves [3,4]. The use of plant raw materials as functional food ingredients is gaining increasing interest among consumers and producers. Common nettle is a valuable source of biologically active substances used in medicine, cosmetics, and textiles [5]. Fresh leaves can be dried and used in powder form in various ways [1]. Leaves harvested before flowering are used for medicinal purposes. Common nettle belongs to the group of phytoalimurgic plants, whose use has increased in recent years due to their positive impact on human health [6].

Over the last decade, the concentration of vehicle pollutants has increased significantly [7,8,9,10] and is one of the main sources of air and soil pollution. According to Singh et al. [11], 90% of air pollution is caused by vehicle emissions, and the pollutants contained in car exhaust gases pose a threat to human health and the environment. Soil enzymatic activity is currently an important tool for assessing the relationship between soil and plants. It is a sensitive indicator of changes in the soil environment compared to physical or chemical properties [12]. Enzymes are biological catalysts involved in the biogeochemistry of elements such as C (carbon), N (nitrogen), P (phosphorus), and S (sulfur). Catalase is an antioxidant enzyme that protects against oxidative stress. It catalyzes the decomposition of hydrogen peroxide into water and oxygen. Dehydrogenase activity is considered an indicator of oxidative metabolism in soil and is associated with many biochemical pathways in soil [13]. Catalase and dehydrogenases belong to the oxidoreductase class of enzymes and are used to obtain information about microbial activity in the soil. Phosphatases (alkaline and acid) play a key role in the mineralization of organic phosphorus bonds [14]. These are the enzymes that react most quickly to environmental stress. *β*-glucosidase is responsible for the final stage of cellulose hydrolysis. It catalyzes the breakdown of disaccharides into glucose available to microorganisms. Proteases catalyze the hydrolysis of peptide bonds in proteins and peptides into amino acids. Alkaline and acid phosphatases, *β*-glucosidase, and proteases are enzymes belonging to the hydrolase class.

Under stressful conditions, metabolic disorders occur in plants, contributing to the excessive production of reactive oxygen species (ROS) [15]. These are natural by-products of metabolism and, if left uncontrolled, can lead to the formation of toxic substances, including hydrogen peroxide (H_2_O_2_) [16]. Plants have the ability to produce preventive and reparative antioxidant mechanisms that protect cells from damage caused by ROS. Preventive mechanisms include antioxidant enzymes such as peroxidase, catalase, and superoxide dismutase, which directly remove ROS. Superoxide dismutase (SOD) catalyzes the conversion of superoxide anions into oxygen and hydrogen peroxide, thus preventing damage. Catalase catalyzes the energy-efficient conversion of H_2_O_2_ into water and oxygen. Repair mechanisms include low-molecular-weight compounds such as vitamins (A, C, E), glutathione, chlorophylls, carotenoids, and phenolic compounds [17].

The study of selected biochemical parameters of plants helps to determine their tolerance to anthropogenic factors. One such index is the air pollution tolerance index (APTI) [18,19,20].

The aim of this study was to determine how different distances from the road traffic affect enzyme activity in soil and the biochemical (enzymatic and non-enzymatic) parameters of nettle leaves used for herbal purposes. The study also attempted to use the air pollution tolerance index (APTI) to determine the level of tolerance of common nettle leaves to pollution depending on the distance from the road traffic.

## 2. Results and Discussion

### 2.1. Activity of Selected Soil Enzymes

**Figure 1:**
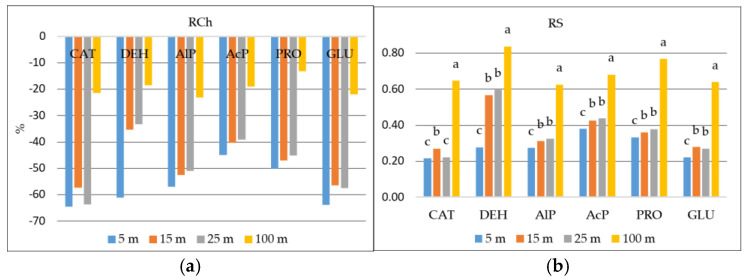
Values of RCh (**a**) and RS (**b**) in soil depending on the distance (5 m, 15 m, 25 m and 100 m) from the road traffic. Different small letters indicate comparisons between distances from traffic at *p* < 0.05.

A one-way analysis of variance showed a significant effect of distance from the road traffic on the activity of selected soil enzymes (Table 1). The activity of the studied oxidoreductase enzymes (CAT and DEH) and hydrolytic enzymes (AlP, AcP, PRO, BG) was highest in the control soil (C) located 1 km from the road. In the case of DEH, AcP, PRO, and BG, no statistical differences in activity were found between soil samples taken 15 m and 25 m from the road. The lowest enzyme activity was obtained in soil samples taken closest to the road (5 m). Based on the results of the RCh coefficient, it was found that this was 65%, 61%, 52%, 45%, 50% and 64% less than at point C for CAT, DEH, AlP, AcP, PRO and BG activity, respectively (Figure 1a). Chaer et al. [21] report that any interference with the natural environment can cause serious changes in the physical, chemical, and biological properties of the soil. This results in a reduction in organic matter, deterioration of its structure, and a decrease in biomass and microbiological and enzymatic activity. The RCh value indicates the direction of anthropogenic pressure (inhibition or activation) on soil enzymatic activity. Similar results were obtained by Hazarika et al. [22], who studied the effect of high-traffic roads on the activity of selected enzymes (polyphenol oxidase, peroxidase, amylase, catalase, dehydrogenase, and urease). According to Li et al. [23], lower CAT activity in soil indicates lower tolerance to oxidative stress. In the case of DEH, however, this is associated with a lower soil respiration rate, which indicates a lower number of microorganisms. Dehydrogenases, a group of oxidoreductases, are crucial in this process. They remove hydrogen atoms from various organic compounds, accelerating the oxidation reactions of organic matter. Lower activity of the tested enzymes involved in C, N, and P biogeochemistry may reduce the rate of organic matter decomposition and the availability of nutrients for plants [24] in the soil along the road, which systematically leads to the degradation of this soil. The long-term reduction in the enzymatic activity of soil exposed to pollution from road traffic consequently leads to a deficiency of nutrients and thus negatively affects plant growth and development [23]. The reduction in the enzymatic activity of soil adjacent to the road at a distance of 5 m may be related to excessive accumulation of heavy metals [22].

When analyzing the impact of the distance between the tested soil and the road traffic on enzyme activity, the resistance coefficient (RS) was used, which is a key concept in monitoring the regeneration of soils affected by anthropogenic pressure [25]. It was found that in soil samples taken at distances of 5 m, 15 m, and 25 m, the resistance of the tested enzymes was significantly lower compared to the activity of enzymes in soil located 100 m away (Figure 1b). By comparing the RS index values between soil enzyme activities, it was determined that their resistance to distance from the road was as follows: DEH > AcP > PRO > AlP > BG > CAT. The lower the RS resistance index values for soil enzymes, the stronger the anthropogenic factor. According to Orwin and Wardle [26], higher RS values indicate maximum resistance of the tested enzymes, and therefore, the impact of the road was lowest in these locations. The RS index value ranges from −1 to +1. A value of 1 means that the distance from the road had no effect on enzyme activity (maximum resistance). In this case, this applies to all enzymes in soils collected from a distance of 100 m (the highest RS values). The lower the values, the stronger the impact of pollutants emitted from the expressway on the enzymes.

The value of the coefficient of determination (R^2^) showed that in the activity of CAT, AlP, AcP, and BG, over 70% of the variability was related to the distance from the expressway (Table 2). Analysis of the regression equation allows us to understand the relationship between the dependent variable and the independent variables. The regression equation showed that increasing the distance from the expressway by 1 m increased CAT activity by 0.005 mg H_2_O_2_ kg^−1^ h^−1^, DEH by 0.0006 mg TPF kg^−1^ 24 h^−1^, AlP by 0.0006 mM pNP kg^−1^h^−1^, AcP by 0.0009 mM pNP kg^−1^h^−1^, PRO by 0.0136 mg TYR kg^−1^h^−1^ and BG by 0.0005 mM pNP kg^−1^h^−1^.

**Figure 2:**
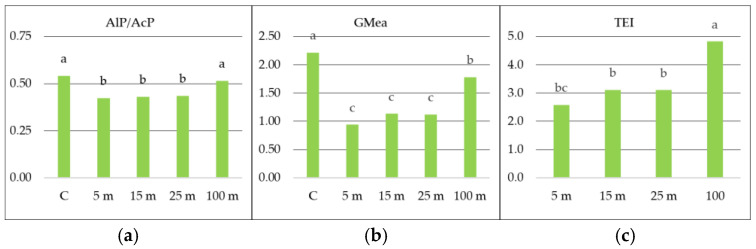
Index of soil enzymes: AlP/AcP (**a**), GMea (**b**), TEI (**c**) in soil depending on the distance (5 m, 15 m, 25 m and 100 m) from the road traffic. Different small letters indicate comparisons between distances from traffic at *p* < 0.05.

Measuring the activity of only one enzyme is not sufficient to determine the impact of environmental factors [27]. Therefore, multiparametric soil quality indicators are used to assess the condition of soil under the influence of abiotic and biotic factors. Based on AlP and AcP activity, an enzymatic indicator of soil pH (AlP/AcP) was calculated (Figure 2a). The AlP/AcP value ranged from 0.42 to 0.54, depending on the distance from the road. According to Dick et al. [28], for optimal plant growth and development, an AlP/AcP value of approximately 0.50 can be considered. In this study, AlP/AcP slightly exceeded 0.5 only for the control (C) and 100 m, but no significant differences were found between these values. For soil 5 m, 15 m, and 25 m away, AlP/AcP was below 0.5, which may indicate acidic soil. Figure 2b shows the GMea index value. Statistically significantly, the highest GMea value was found in the control soil (C) (2.21) and 100 m (1.78) away from the road traffic. According to Jat et al. [29], the value of this indicator is related to the physicochemical and biological properties of the soil. Lower GMea values in soil located 5 m, 15 m, and 25 m away from the road traffic indicate lower soil quality and may describe changes in soil quality, disregarding physicochemical properties [12]. According to Jarosz et al. [30], GMea can be used as an indicator of soil biological quality. The total enzyme activity (TEI) index was used to assess the total enzymatic activity of the soil. It was found that the change in the value of this indicator was significantly dependent on the distance from the road traffic (Figure 2c). The highest TEI value was obtained in the control soil (C) (9.22). In soil 100 m away from road traffic, the TEI value was significantly lower by 20% (7.41). No significant differences in TEI were found between the values at 5 m, 15 m, and 25 m. According to Tan et al. [31], TEI facilitates the comparison of the combined enzyme activity and quality of each soil sample. The direction of change in GMea and TEI values was similar. The results of Jarosz et al. [30] indicated that GMea was more sensitive than the TEI index.

### 2.2. Biochemical Parameters in Common Nettle Leaves

Vehicle emissions are the main factor contributing to air pollution [32]. Toxins released by vehicles are absorbed by plants growing along roads. This causes biochemical changes in both cultivated and wild plants. The content of photosynthetic pigments in common nettle leaves was statistically significantly dependent on the distance from the emitter (Table 3). The lowest content of both Chl a (0.642 mg g^−1^ F.W.) and Chl b (0.223 mg g^−1^ F.W.) was obtained in samples of common nettle leaves collected 5 m from the road traffic. This was 58% and 62% less, respectively, compared to the control (C). The breakdown of chlorophyll pigments is a measurable response of plants to stressful conditions [33]. The regression equation (Table 3) showed that with each 1 m increase in distance from the highway, the Chl a content will increase by 0.0024 mg g^−1^ F.W. and Ch b by 0.0003 mg g^−1^ F.W.

Carotenoids, like chlorophylls, are secondary metabolites and antioxidants that neutralize free radicals and other reactive oxygen species, protecting cells from oxidative damage [34]. Car (carotenoid) content in common nettle leaves was dependent on different distances from the road traffic (Table 3). Increasing distance from the road traffic significantly increased Car content in leaves compared to the control sample (C). A positive correlation (r = 0.793) was observed between distance from the road and Car content (Table 4). The R^2^ value explained 62.3% of the variability in Car dependent on distance from the road traffic, the remaining 37.7% by other factors. According to Neves et al. [35], road dust plays a significant role in reducing carotenoid content in plants. Road dust has a low pH, which can lead to chlorophyll degradation [36].

**Figure 3:**
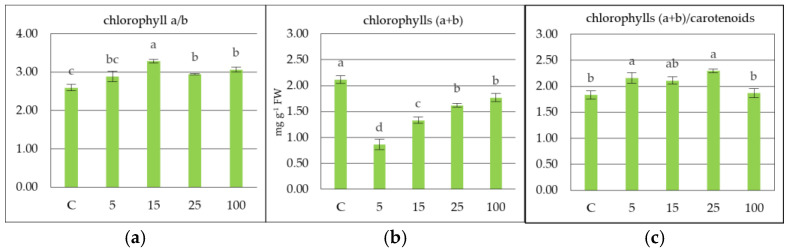
The value of the chlorophyll a/b ratio (**a**), the sum of chlorophyll a and b (a+b) (**b**), and the value of the chlorophyll a+b/carotenoids ratio (**c**), depending on the distance from the road traffic. Different small letters indicate comparisons between distances from traffic at *p* < 0.05; ±standard deviation.

Based on the results of the content of the tested plant pigments, the values of the Chl a/b ratio, the sum of Chl a+b, and the Chl a+b/Car ratio were calculated (Figure 3a–c). The Chl a/b indicates the relative proportion of Chl a to Chl b. The leaves of common nettle collected from location C showed a lower ratio compared to the other locations within the emitter’s range of influence (Figure 3a). This indicates a relatively higher proportion of Chl b in the total chlorophyll content. Chl a and b may react differently to stress factors [37]. The total chlorophyll content (Chl a+b) is an indicator of photosynthetic activity. It was observed that the Chl a+b value increases with distance from the road, which may be related to a decrease in pollution load (Figure 3b). Low Chl a+b content in leaves under high stress conditions is associated with low plant tolerance. The (Chl a+b)/Car ratio was significantly higher in samples of common nettle leaves collected from points 5 m and 25 m from the road traffic, suggesting an increased proportion of total chlorophyll relative to Car (Figure 3c) Sonobe et al. [38]. The results indicate that the growth and development of nettles along a highway may lead to changes in the content of Chl a, Chl b, Car, and the mutual ratio of these pigments. This reflects biochemical reactions to air pollution depending on the distance from the road traffic.

The average AAC content in common nettle leaf samples is presented in Table 3. The AAC content in leaves collected at distances of 5 m, 15 m, 25 m, and 100 m was significantly lower than at site C. This content ranged from 5.775 mg 100 g^−1^ F.W. to 12.17 mg 100 g^−1^ F.W. The lowest content was obtained in samples from a distance of 5 m (5.775 mg 100 g^−1^ F.W.). No statistical difference was found between the AAC content in samples from 15 m and 25 m. ACC is an antioxidant that affects plant resistance to stress factors, promoting tolerance to pollution [39]. However, the low AAC content in areas most exposed to road dust pollution can be explained by the consumption of AAC during the removal of cytotoxic ROS after the penetration of pollutants into the tissues of common nettle leaves [19]. The AAC content in plants can range from 16.00 to 112.8 mg 100 g^−1^ FW or even up to 238 mg 100 g^−1^ FW [40], and this depends on both natural and anthropogenic factors. A statistically significant positive correlation (r = 0.752) was found between the distance from the road and the AAC content in common nettle leaves (Table 4). Based on the regression equation, it was found that with each 1 m increase in distance from the road, the AAC content will increase by 0.0076 mg g^−1^ FW.

The pH value of the common nettle leaf extract was significantly determined by the distance from the road traffic (Table 3). The pH of common nettle leaf extracts ranged from 4.05 to 5.5. The highest pH values were obtained in nettle leaves collected at location C (5.50) and 100 m (5.42) from the road. No significant differences were found between them, similar to common nettle leaves from points 15 m and 25 m. Significantly, the lowest value (4.05) was obtained closest to the road traffic (5 m). Rai and Panda [39] and Skrynetska et al. [41] also showed that the pH values of plant extracts from roadside verges were acidic. Mehmood et al. [20] found that a change in the pH of the extract indicates the plant’s response to stress, as this parameter is closely related to the process of photosynthesis or enzymatic activity [42]. Higher pH values also increase the production of ascorbic acid.

Relative water content (RWC) was found to change significantly with distance from road traffic (Table 3). Under the influence of stress factors, RWC decreases [20]. Higher water content in plants helps maintain their physiological and biochemical balance under polluted conditions. In our study, RWC in common nettle leaves collected 5 m from the road was the lowest (45%), and with increasing distance, RWC statistically increased. This is confirmed by the results of Skrynetska et al. [41], who studied *Plantago major* and *Plantago lanceolata*.

As shown in Table 3, distance from the traffic source had a significant effect on SOD and CAT activity in common nettle leaves. Compared to control values (SOD 35.96 U g^−1^ FW; CAT 6.235 mg H_2_O_2_ kg^−1^ h^−1^), the activity of oxidoreductive enzymes was significantly highest in common nettle leaves collected 5 m from the road traffic. This was a decrease of 320% and 101%, respectively. For both enzymes, no significant differences in activity were found at points 5 m and 15 m away from the road. According to Jeddi et al. [43], plants produce antioxidant enzymes as defense mechanisms to combat oxidative stress. Increased activity of the studied antioxidant enzymes is associated with intracellular H_2_O_2_ levels. These enzymes capture harmful H_2_O_2_, inhibiting oxidative bursts induced by road pollutants. Excess O_2_ under stress conditions can be converted to H_2_O_2_ by SOD. It is then metabolized by, among others, CAT [44]. Therefore, an increase in antioxidant enzyme activity is believed to be associated with stress resistance. According to Shen et al. [45], SOD and Car are the two most effective of the nine major antioxidants (ascorbate, glutathione, polyamines, α-tocopherol, carotenoids, catalases, ascorbate peroxidase, superoxide dismutase, and glutathione-S-transferase). Studies by Mukhopadhyay et al. [42] showed that the reduction in chlorophyll content and antioxidant enzyme activity in *Murraya paniculata* could be caused by PAHs in industrial dust. Our study did not include an assessment of the contaminant content in dust or soil. Therefore, further studies on the effects of selected contaminants on plants are necessary.

The values of the coefficient of determination (R^2^) were lowest for CATp and SOD activity, pH, and AAC content: 55.5%, 59.9%, 52.1%, and 56.5%, respectively (Table 4). This means that 45.2%, 44.5%, 40.1%, 47.9% and 43.5% of these parameters were influenced by factors other than distance from the road traffic.

**Figure 4:**
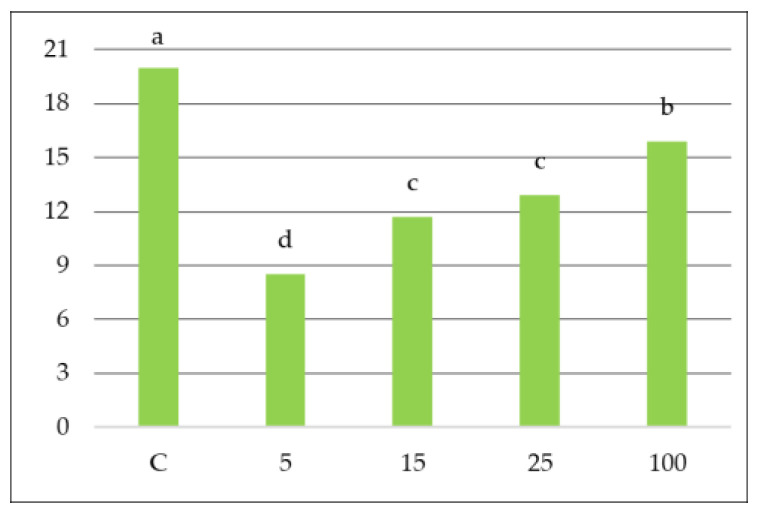
APTI (air pollution tolerance index) values in common nettle leaves depending on distance from the road traffic. Different small letters indicate comparisons between distances from traffic at *p* < 0.05; ± standard deviation.

To assess the sensitivity or tolerance of common nettle to air pollution, the results of total chlorophyll content (Chl a + b), AAC, leaf extract pH, and RWC were used to calculate the air pollution tolerance index (APTI) [41,46]. Figure 4 shows APTI values depending on the distance from the road. Common nettle samples taken from locations 5 m, 15 m, 25 m, and 100 m from the road were sensitive to pollution (APTI: 8.51, 11.68, 12.91, and 15.91, respectively). In contrast, common nettles collected at point C were characterized by an average (19.97) sensitivity to air pollution. These values can be explained by exposure to pollution from roads, as a result of which *Urtica dioica* L. in the study group (5 m–100 m) shows lower antioxidant defense system effectiveness compared to the control group. It was found that the location of plant sampling, depending on the distance from the expressway, significantly influenced the APTI value. According to Nagórska-Socha et al. [18] and Enitan et al. [19], the APTI of the same plant may vary depending on the location due to different levels of air pollution.

**Figure 5:**
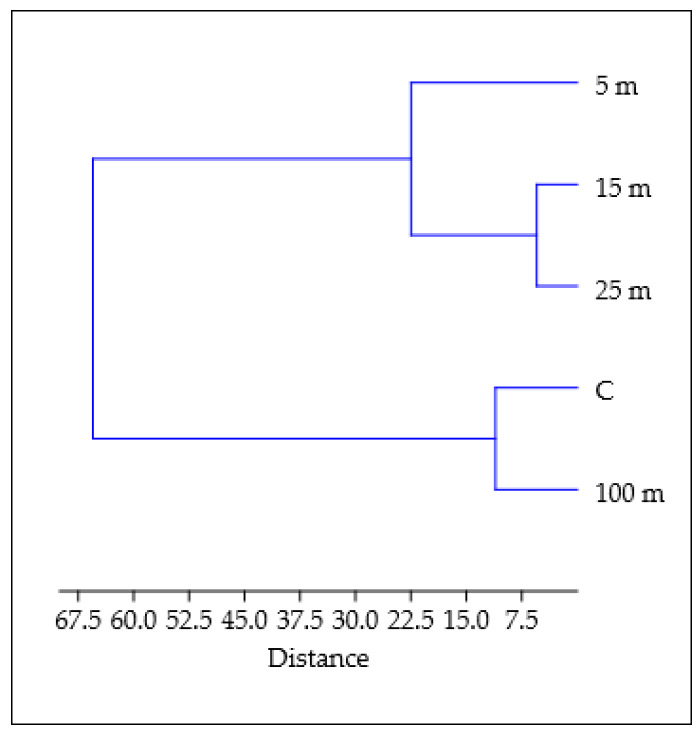
Cluster analysis determined based on the activity of selected soil enzymes and biochemical parameters of common nettle depending on distance from the road traffic.

The dendrogram (Figure 5) showing the proximity of connections between locations from which both soil samples and common nettle leaves were collected was created based on a cluster analysis performed based on Euclidean distances between features and Ward’s agglomeration method [47]. Reading the graph from the closest to the furthest neighborhood, it can be concluded that the greatest similarity is between points 15 m and 25 m. Next, point 5 m is similar to these two features. Samples taken from a location 100 m away and the control (C) were also similar, but the Euclidean distances were slightly greater between the tested parameters compared to 15 m and 25 m.

## 3. Materials and Methods

### 3.1. Soil and Plant Sampling Location

In order to determine the impact of national road No. 10 (DK10) on the activity of selected soil enzymes from the oxidoreductase and hydrolase classes and selected biochemical parameters in the leaves of common nettle, in 2023, samples were collected for testing from natural habitats located at various distances from road traffic (5 m, 15 m, 25 m, 100 m, and control) in Pawłówek (53°09′; 17°51′ E) in the Bydgoszcz district, in the municipality of Sicienko, on the Nakło-Pawłówek section. In this area, the temperature ranges from −4 °C to 24 °C throughout the year and rarely falls below −14 °C or exceeds 30 °C. Rainfall occurs throughout the year. The rainiest month is July, when the average rainfall is 58 mm. National Road No. 10 (DK10) is a transport route in Poland, connecting Bydgoszcz with Toruń and Warsaw, running through the provinces of West Pomerania, Greater Poland, Kuyavia-Pomerania, and Masovia. The total length of DK10 is 467 km, most of which has GP class parameters (GP class road, main expressway (colloquially, expressway)), i.e., a main road for accelerated traffic. The average daily annual traffic (SDRR) on the Nakło-Pawłówek section is 11,253 vehicles, including 2455 heavy vehicles. SDRR is a measure of traffic intensity. It determines the average number of vehicles that pass through a given section of road in 24 h, calculated on an annual basis.

### 3.2. Activity of Selected Soil Enzymes

Soil samples were collected from the roadside using an auger at depths ranging from 0 to 15 cm. Common nettle leaves were also collected. For comparative analysis, samples were also taken from a control point (C), which had less noticeable traffic intensity. Soil and nettle samples were collected for analysis in five replicates at each sampling point (totaling 25 soil and 25 nettle samples). Plant samples were pooled and considered representative of each sampling site. The soil samples were treated similarly. Samples were collected in accordance with the PN-R-04031:1997 standard [48] for collecting and preparing soil samples for chemical and agricultural analysis.

Enzyme activity was determined in fresh sieved (<2 mm) soils.

- Catalase (CATs) was determined using the Johnson and Temple method [49] with a 0.3% hydrogen peroxide solution as substrate. The remaining H_2_O_2_ was determined by titration with 0.02M KMnO_4_ under acidic conditions.
- Dehydrogenases (DEH) activity was determined using the Thalmann method [50] after incubating the sample with 2,3,5-triphenyltetrazolium chloride and measuring the absorbance of triphenylformazan (TPF) at 546 nm and expressed in mg TPF kg^−1^ 24 h^−1^.
- Alkaline phosphatase (AlP) and acid phosphatase (AcP) activity was determined based on the detection of p-nitrophenol (pNP) released after incubation (37 °C, 1h) at pH ~6.5 for acid phosphatase and pH~11.0 for alkaline phosphatase [51].
- Protease activity was determined using the Ladda and Butler method [52], where the concentration of the amino acid tyrosine (Tyr) was determined in soil samples after incubation with sodium caseinate. Absorbance was measured with a spectrophotometer at λ = 680 nm.
- *β*-glucosidase (BG) activity was measured using the Eivazi and Tabatabai method [53], using p-nitrophenyl-*β*-D-glucopyranoside as a substrate. The concentrations of p-nitrophenol were determined by direct reading of the sample at 400 nm after alkalization with Tris/NaOH buffer (pH 10.0) and CaCl_2_.

Soil quality indicators were calculated based on enzyme activity:

- The actual activity value was given as a relative change (RCh) in relation to the control soil (C) [21]:
  (1) $$RCh=\left(\frac{P_{0}}{C_{0}}-1\right)\times 100$$
- Soil resistance index (RS) is calculated using the formula proposed by Orwin and Wardle [26]:
  (2) $$RS=1-\left[\frac{2\left|D_{0}\right|}{\left(C_{0}+\left|D_{0}\right|\right)}\right]$$
  for both indicators: $D_{0}=C_{0}-P_{0}$, $C_{0}$—enzyme activity in the control soil; $P_{0}$—enzyme activity in soil exposed to traffic (5 m, 15 m, 25 m and 100 m). RS is a dimensionless indicator. The RS value is expressed as a ratio of two quantities with the same units and therefore has no unit of measurement.
- Enzymatic pH indicator [28]:
  (3) AlP/AcP
- Geometric mean GMea [54]:
  (4) $$GMea=\sqrt[{6}]{CAT\times DEH\times AlP\times AcP\times PRO\times BG}$$
- The total activity of soil enzymes (TEI) (total enzyme activity index) was calculated as follows [31]:
  (5) $$TEI=\sum \frac{Xi}{\bar{Xi}}$$
  where Xi is the activity of soil enzyme *i*, and $\bar{Xi}$ is the mean activity of enzyme *i* in all the samples.

### 3.3. Biochemical Analysis of Common Nettle Leaves

Plant samples for laboratory analysis were collected completely at random at five locations at each of the distances from the road studied. The following were identified in fresh leaves of common nettle:

- The content of chlorophyll a (Chl a) and chlorophyll b (Chl b) and carotenoids was determined according to Lichtenthaler [55] and Lichtenthaler and Buschmann [56]. A spectrophotometer was used to measure the content of chlorophyll a, chlorophyll b, and carotenoids at wavelengths (λ max) of 645 nm, 662 nm, and 470 nm, respectively. Based on the Chl a and Chl b content, the ratio of these pigments (Chl a/b), which is an indicator of leaf health, and the total chlorophyll content (a+b) were determined. The value of the ratio Chl (a+b)/Car was also calculated.
- The content of ascorbic acid (AAC) was determined by titration in an acidic medium with a standard solution until a pink color appeared [57].
- The pH of the leaves was determined potentiometrically after homogenizing 5 g of plant material in 10 mL of deionized water [39].

To measure the relative water content (RWC) of common nettle leaves, the leaves were weighed to obtain their fresh weight (FM). They were then placed in test tubes filled with water and left in the dark for 24 h. After this time, they were weighed again to determine the mass at full turgor (TM). The leaves were then left for 48 h to dry completely at 65 °C and weighed again to obtain their dry mass (DM) [39]. The RWC was then calculated using the following formula:

(6) $$RWC\left(\%\right)=\frac{FM-DM}{TM-DM}\times 100\%$$

- Catalase activity (CATp) was determined according to the method of Kar and Mishra [58].
- Superoxide dismutase (SOD) activity was determined according to the method of Beauchamp and Fiodorovich [59], in which the measure of enzymatic activity is the ability to inhibit the photochemical reduction in tetrazolium blue. Absorbance value at a wavelength of 560 nm.
- Based on four biochemical parameters (AAC, Chl a+b, pH and RWC) in common nettle leaves, the air pollution tolerance index (APTI) was calculated according to Prajapati and Tripathi [46].
  (7) $$APTI=\frac{AAC\times [\left(\mathrm{Chl}a+b)+\left.pH\right]\right)+RWC}{10}$$
  where AAC is ascorbic acid, Chl a+b is total chlorophyll content, pH is leaf extract pH, and RWC is relative water content. The tolerance range of plants is as follows: <1—very sensitive; 1–16—sensitive; 17–29—moderately sensitive; 30–100—resistant to air pollution.

### 3.4. Statistical Analysis

The results obtained were subjected to statistical analysis, which was performed in MS Excel and Statistica 13.3. The normalization of the empirical data distribution was performed using the Shapiro–Wilk test of normality. A one-way analysis of variance was performed using Duncan’s post hoc HSD test for a significance level of *p* = 0.05 to determine significantly different objects. The results are presented as arithmetic mean and standard deviation (±SD). For the relationship between soil enzyme activity (CATs, DEH, AlP, AcP, PRO, and BG), biochemical non-enzymatic parameters (Chl a, Chl b, Car, AAC, AA, pH, RWC), selected enzymatic markers of plant resistance (CATp and SOD), and distance from the expressway, the Pearson linear correlation coefficient r was used. Regression analysis was also used to examine the relationship between the dependent variable (the tested soil enzymes and selected parameters of common nettle) and the independent variable (distance from the road). The value of the coefficient of determination (R^2^) was used, which determines how much of the variability of the dependent variable is explained by the independent variable in the regression model. Multivariate cluster analysis according to Ward’s method [47] was also used to minimize variance within groups. It allows data to be divided into clusters so that objects within a single cluster are as similar as possible to each other and, at the same time, as different as possible from objects in other clusters. The analysis was performed using the PAST 4.13 program [60].

## 4. Conclusions

The presented enzyme activity results demonstrate that this parameter is a valuable tool for assessing soil quality. Lower enzyme activity in the soil closest to the expressway compared to the control indicates soil degradation. Changes in soil enzyme activity with distance from the expressway result in both non-enzymatic and enzymatic biochemical changes in common nettle. Air pollution tolerance index values indicate that the ability of common nettle to tolerate pollution depends on biochemical processes occurring both in the soil and within the plant itself. The results of the air pollution tolerance index show that common nettle growing along an expressway, regardless of its distance, is sensitive to road pollution. Therefore, its raw use for medicinal or cosmetic purposes is not recommended. The results of this study may provide valuable information on the quality of common nettle leaves growing wild along roads and their potential use for phytotherapeutic purposes.

## Figures and Tables

**Table 1:** Activity of selected oxidoreductive and hydrolytic enzymes in soil depending on the distance from the road traffic.

Enzymes	Distance				
	C	5 m	15 m	25 m	100 m
CATs	0.935 ^a^ ± 0.0276	0.332 ^d^ ± 0.0134	0.398 ^c^ ± 0.0042	0.340 ^d^ ± 0.0021	0.734 ^b^ ± 0.0269
DEH	1.411 ^a^ ± 0.002	0.549 ^e^ ± 0.0191	0.912 ^c^ ± 0.0042	0.942 ^c^ ± 0.0148	1.149 ^b^ ± 0.0573
AlP	1.261 ^a^ ± 0.0160	0.542 ^e^ ± 0.0085	0.598 ^d^ ± 0.0086	0.618 ^c^ ± 0.0078	0.970 ^b^ ± 0.0163
AcP	2.327 ^a^ ± 0.0262	1.280 ^e^ ± 0.0127	1.389 ^c^ ± 0.0042	1.416 ^c^ ± 0.0057	1.884 ^b^ ± 0.0170
PRO	32.31 ^a^ ± 0.7849	16.14 ^c^ ± 0.2687	17.11 ^c^ ± 0.0990	17.74 ^c^ ± 0.7212	28.06 ^b^ ± 0.5586
BG	0.939 ^a^ ± 0.0212	0.340 ^d^ ± 0.0163	0.410 ^c^ ± 0.0035	0.399 ^c^ ± 0.0049	0.733 ^b^ ± 0.0290

Abbreviations: C—control; CATs—catalase (mg H_2_O_2_ kg^−1^ h^−1^); DEH—dehydrogenases (mg TPF kg^−1^ 24 h^−1^); AlP—alkaline phosphatase (mM pNP kg^−1^h^−1^); AcP—acid phosphatase (mM pNP kg^−1^h^−1^); PRO—protease (mg TYR kg^−1^h^−1^); BG—*β*-glukosidase (mM pNP kg^−1^h^−1^). Different small letters indicate comparisons between distances from traffic at *p* < 0.05; ± standard deviation.

**Table 2:** Regression summary for dependent variables (y) calculated for soil enzymes depending on distance from the road traffic.

Equation	r	R^2^ (%)	*p*
* CATs = 0.4262 + 0.005x	0.843	71.0	0.0730
DEH = 0.8612 + 0.0006x	0.779	60.7	0.1205
AlP = 0.6536 + 0.0006x	0.882	77.9	0.0476
AcP = 1.4522 + 0.0009x	0.892	79.6	0.0476
PRO = 19.0975 + 0.0139x	0.812	65.9	0.0420
BG = 0.4464 + 0.0005x	0.854	73.0	0.0950

* See Table 1.

**Table 3:** Selected biochemical parameters in common nettle leaves depending on the distance from road traffic.

Parameters	Distance				
	C	5 m	15 m	25 m	100 m
Chl a	1.528 ^a^* ± 0.0283	0.642 ^e^ ± 0.0141	1.021 ^d^ ± 0.0502	1.210 ^c^ ± 0.0021	1.333 ^b^ ± 0.0078
Chl b	0.589 ^a^ ± 0.0269	0.223 ^d^ ± 0.0064	0.311 ^c^ ± 0.0057	0.411 ^b^ ± 0.0064	0.435 ^b^ ± 0.0240
Car	1.156 ^a^ ± 0.0438	0.400 ^e^ ± 0.0127	0.631 ^d^ ± 0.0042	0.706 ^c^ ± 0.0092	0.947 ^b^ ± 0.0410
ACC	15.78 ^a^ ± 0.2899	5.775 ^d^ ± 0.1485	8.085 ^c^ ± 0.1768	9.040 ^c^ ± 0.1131	12.17 ^b^ ± 0.1131
pH	5.50 ^a^ ± 0.005	4.05 ^c^ ± 0.002	4.75 ^b^ ± 0.006	4.92 ^b^ ± 0.009	5.42 ^a^ ± 0.006
RWC	72 ^a^ ± 0.259	45 ^d^ ± 0.158	52 ^c^ ± 0.189	58 ^b^ ± 0.186	68 ^a^ ± 0.208
SOD	35.96 ^d^ ± 1.082	72.30 ^a^ ± 0.9334	65.02 ^b^ ± 1.131	61.99 ^b^ ± 0.3465	43.26 ^c^ ± 0.3748
CATp	6.235 ^d^ ± 1.4849	26.17 ^a^ ± 0.0778	21.14 ^b^ ± 0.5798	22.97 ^b^ ± 0.8415	9.100 ^c^ ± 0.4808

C—controle; Chl a—chlorophyll a (mg g^−1^ F.W.); Ch b—chlophyll b (mg g^−1^ F.W.); Car—carotenoids (mg g^−1^ FW); AAC—ascorbic acid content (mg 100 g^−1^ F.W.); ARA—antiradical activity (%); RWC—relative water content (%); SOD—superoxide dismutase (U g^−1^ FW); CATp—catalase (mg H_2_O_2_ kg^−1^ h^−1^); * see Table 1.). Different small letters indicate comparisons between distances from traffic at *p* < 0.05; ± standard deviation.

**Table 4:** Regression summary for dependent variables (y) calculated for selected biochemical parameters of common nettle leaves depending on distance from the road traffic.

Equation	r	R^2^ (%)	*p*
* Ch a = 3.5435 + 0.0024x	0.651	72.4	0.2342
Chl b = 0.3333 + 0.0003x	0.827	68.5	0.0838
Car = 0.6457 + 0.0005x	0.793	62.3	0.1095
AAC = 8.4223 + 0.0076x	0.752	56.5	0.0672
pH = 7.5283 + 0.0056x	0.722	52.1	0.0126
RWC = 18.3588 + 0.0135x	0.781	60.9	0.0589
CATp = 20.6141 − 0.0153x	−0.745	55.5	0.1484
SOD = 61.9933 − 0.0275x	−0.774	59.9	0.1245

* see Table 3.

## Data Availability

The raw data supporting the conclusions of this article will be made available by the authors on request.
